# Application of Novel Wearable Self-Balancing Exoskeleton Robot Capable for Complete Self-Support in Post-stroke Rehabilitation: A Case Study

**DOI:** 10.7759/cureus.63831

**Published:** 2024-07-04

**Authors:** Yanzheng Zhang, Zhanhe Li, Yuanyuan Zhang, Yefan Cao, Lei Li, Hewei Wang

**Affiliations:** 1 Department of Rehabilitation Medicine, Shanghai Hebin Rehabilitation Hospital, The Affiliated Hospital of Shanghai Huashan Hospital, Shanghai, CHN; 2 Department of Rehabilitation Medicine, Huashan Hospital, Fudan University, Shanghai, CHN; 3 Department of Rehabilitation Medicine, The First Affiliated Hospital of Shandong First Medical University, Jinan, CHN

**Keywords:** rehabilitation, lower limb function, balance, wearable self-balancing exoskeleton robot, stroke, case report

## Abstract

Early weight-bearing and trunk control training are essential components for promoting lower limb motor recovery in individuals with stroke. In this case study, we presented the successful implementation of a three-week wearable self-balancing exoskeleton robot training program for a 57-year-old male patient who had suffered from a stroke. After carefully reviewing the patient's previous medical records, conducting a thorough assessment, and excluding other potential contraindications, we introduced wearable self-balancing exoskeleton robot training to complement conventional rehabilitation in managing balance and lower limb function. The training program included early initiation of weight bearing and trunk control training following an ischemic stroke, aimed at promoting motor recovery and improving functional independence. The findings indicated that training with a wearable self-balancing exoskeleton robot enhanced the balance and motor function of the hemiplegic patient, with commendable adherence. Furthermore, the participants consistently reported increased satisfaction and confidence during the training sessions. This case report not only provided preliminary evidence of the effectiveness of the wearable self-balancing exoskeleton robot in promoting functional recovery following a stroke but also outlined a comprehensive training program that may hold value for future clinical application.

## Introduction

Walking problems occur in up to 80% of persons post-stroke, and it is reported that 70% of individuals with stroke are at risk for falling [1]. Enhancing the quality of life and promoting psychological well-being for stroke survivors relies on achieving safe, independent, effective, and efficient real-world mobility. This not only improves functional independence but also fosters self-reliance [2].

The 2016 American Heart Association/American Stroke Association Guideline recommends robot-assisted movement training to improve motor function after stroke as class IIb-level A evidence [3], and it is also endorsed as level A evidence by the 2019 Canadian Stroke Best Practice Recommendations [4]. One meta-analysis shows that patients with severe lower limb impairment post-stroke demonstrate better outcomes in terms of walking movements and daily activities when they receive robot-assisted rehabilitation [5]. With the assistance of a robot, many recommended training, such as early initiation of weight-bearing, integration of trunk and limb activities, controllable balance training, and attainment of a symmetrical gait pattern, can be feasibly implemented with high dosage and prolonged duration [6]. The use of robot training conserves significant therapist resources and reduces the time for participants to commence recovery, potentially enabling individuals with latent capabilities to achieve their maximum potential for rehabilitation.

The self-balancing exoskeleton robot is an innovative technology that enables participants’ weight-bearing training even in the stage of paralysis. It can also elicit protective reactions to maintain balance when shifting the center of gravity, thereby promoting the restoration of trunk balance control function. Furthermore, with this technology, therapists can now focus on therapeutic intervention while minimizing falling risks. A user satisfaction study recruited a group of seven individuals with physical disabilities and found that the self-balancing exoskeleton robot demonstrated superior ease of transfer compared to other robotic devices [7], thus offering significant rehabilitation opportunities for persons with severely impaired locomotor function. 

Currently, while the effectiveness of robots in lower limb stroke rehabilitation has been recognized; however, the actual application modalities in clinical practice vary, and there is a lack of training programs with sufficient details. Therefore, this case study aims to demonstrate the utilization of wearable self-balancing exoskeleton robots in the balance and lower limb function rehabilitation of stroke patients, with a specific focus on its clinical application methods and clinical efficacy. This case report adheres to the CAse REport (CARE) guidelines.

## Case presentation

Patient information

**Figure 1:**
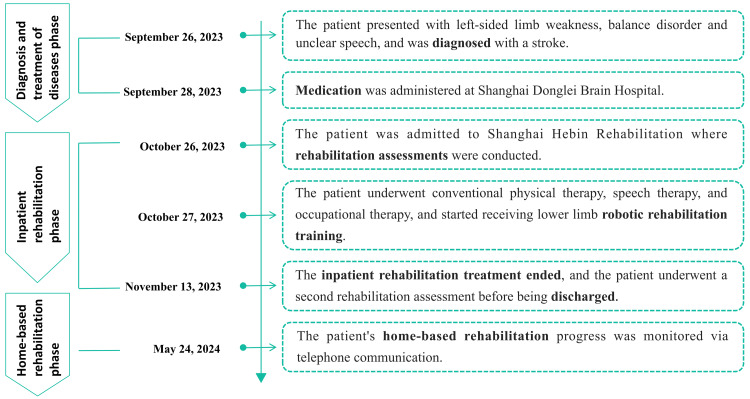
Timeline. The figure shows the critical time points of the patient for diagnosis, treatment, rehabilitation, and follow-up from September 26, 2023, to May 24, 2024.

A 57-year-old male was admitted to the rehabilitation hospital due to left-sided weakness, balance disorder, and slurred speech one month ago on September 26, 2023. After being diagnosed with an ischemic stroke (infarction in the right centrum semiovale and genu of corpus callosum, as well as bilateral subcortical lacunar infarctions), he received drug treatment in the hospital, including clopidogrel bisulfate tablets, atorvastatin calcium tablets, and other medications to improve and circulation and restore cerebral perfusion. Although his condition stabilized after the acute phase of stroke treatment, the movement disorder still significantly impacted his daily life. The patient was capable of sitting independently but required assistance for standing, walking, and performing basic self-care tasks, which caused significant distress. The patient had a 20-year history of diabetes but achieved acceptable blood glucose control with subcutaneous insulin and oral metformin. He does not have any significant family medical history or orthopedic conditions, joint diseases, or other complications. Moreover, the patient demonstrated clear consciousness without any cognitive impairment and exhibited the ability to comprehend and successfully execute medical staff instructions. He received conventional physical therapy, speech therapy, and occupational therapy subsequently. The patient was informed by the rehabilitation therapist about the availability of a wearable self-balancing exoskeleton robot that could provide a stable standing support platform to promote the recovery of balance and lower limb function. Recognizing the potential benefits, the patient proactively chose this treatment method on October 27, 2023, driven by a strong desire for prompt recovery (Figure 1). 

Clinical findings

The Brunnstrom recovery stage assessment showed that the patient's left upper limb, hand, and lower limb were classified as stage III, stage III, and stage II, respectively. The hemiplegic limbs did not show spasticity, and the Modified Ashworth Scale (MAS) score was 0. The simplified three-level balance assessment method revealed that the patient exhibited a level 2 sitting balance (capable of sustaining dynamic balance for over three seconds, accompanied by upper limb movement) and a level 0 standing balance (incapable of maintaining static self-balance for more than three seconds). In daily life, the patient heavily relied on wheelchairs for his activities. The patient reported no pain during daily activities and rehabilitation treatment. The patient exhibited hyporeflexia and positive pathological reflexes or hyper-reflexia in the affected lower limb (Table 1). The patients had normal body size, with 168 cm height and 66 kg weight. The assessment results of the active and passive range of motion in the upper and lower limbs were presented in Table 2, indicating that the patient fulfilled the fundamental criteria for undergoing robotic training.

**Table 1:** Reflexes. (+): hypotonia; (++): normal.

Reflexes	Left (affected side)	Right (unaffected side)
Knee jerk (L_3-4_)	+	++
Ankle jerk (S_1_)	+	++
Babinski	Positive	Negative

**Table 2:** Range of motion evaluated prior to the administration of training using the wearable self-balancing exoskeleton robot. AROM: active range of motion; PROM: passive range of motion.

	Joint	Left side (AROM, degree)	Left side (PROM, degree)
Upper limb	Shoulder flexion	0-60	0-130
	Shoulder abduction	0-50	0-110
	Elbow flexion	0-130	0-130
Lower limb	Hip flexion	0	0-110
	Hip abduction	0	0-40
	Ankle plantar flexion	0	0-35
	Ankle dorsiflexion	0	0-10

Diagnostic assessment

**Figure 2:**
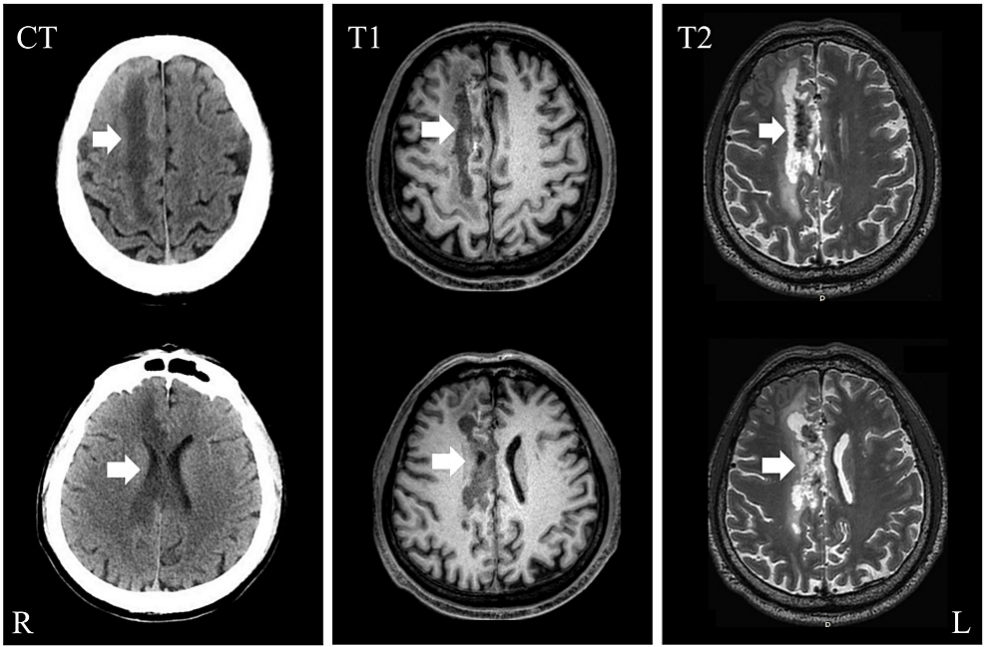
CT and MRI (T1&T2) imaging. The computed tomography scan revealed the presence of infarctions in the right parietal lobe and corpus callosum, accompanied by a small local hemorrhagic lesion. The MRI findings showed infarctions in the right centrum semiovale and genu of corpus callosum, as well as bilateral subcortical lacunar infarctions.

The computed tomography scan revealed the presence of a right parietal lobe and corpus callosum infarction, accompanied by a small local hemorrhagic lesion. The MRI findings revealed infarction in the right centrum semiovale and genu of corpus callosum, as well as bilateral subcortical lacunar infarctions (Figure 2).

Therapeutic interventions

When the patient arrived at our hospital for rehabilitation treatment, he was already in the subacute stage of stroke. During this period, the general condition of the patient gradually stabilized, and there were varying degrees of functional impairments. Therefore, comprehensive and systematic rehabilitation treatment was required. Specifically, the patient underwent an intensive conventional rehabilitation program tailored to address the specific problems identified during the assessment (Table 3).

**Table 3:** Conventional rehabilitation.

Problem identified	Goal	Intervention
Long-term bedridden	To prevention venous thrombosis	Air pressure wave therapy
Muscle atrophy	To reduce muscle atrophy and enhance muscle strength	Electrical stimulation to the paralyzed muscles of both upper and lower limbs on the affected side; Progressive cycling training using a stationary bike (MOTOmed), transitioning from passive to assisted, and ultimately active training modes
Limitations in activities of daily living	To restore self-care ability	Occupation-based and task-oriented activities of hand and upper extremities addressing fine and gross motor functions
Proprioception deficits	Proprioceptive input	Electric standing bed training; vibration therapy
Impairment in mobility	To improve balance and walking ability	Symmetrical weight bearing between lower limbs in stance; weight shifting training; sit-to-stand training; stepping training; functional reaching training

**Figure 3:**
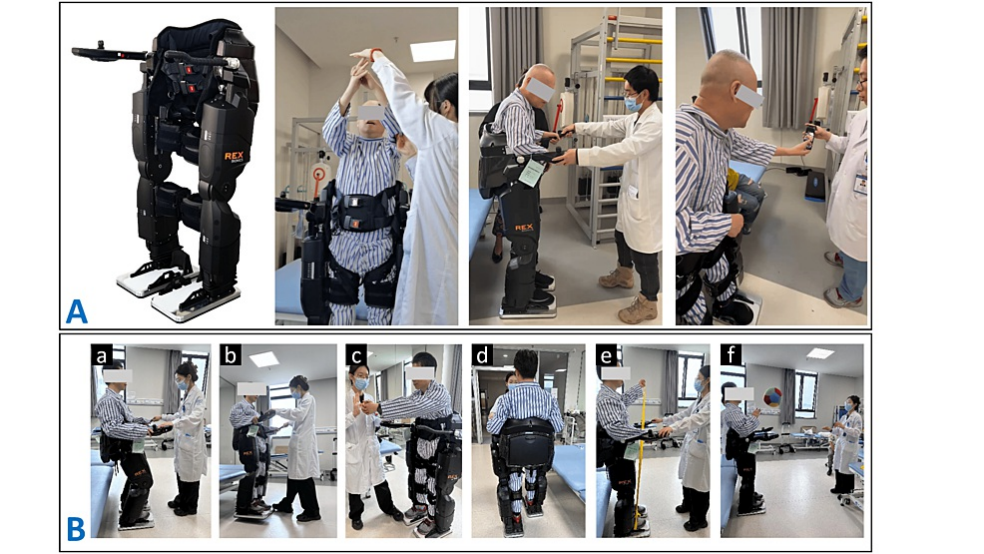
Application of wearable self-balancing exoskeleton robot for balance and lower limb function in stroke patients. (B-a): Sit-to-stand training; (B-b): Weight-bearing training for the hemiplegic leg; (B-c): Trunk control training; (B-d): Walking ability training; (B-e): Resistance training for upper limbs; (B-f): Functional training for upper limbs.

In addition, a specialized training program utilizing a wearable self-balancing exoskeleton robot (REX-L, MaxRex Robotic Exoskeleton Ltd., Wuxi, Jiangsu, China) was developed to enhance the patient's balance and lower limb motor function. Different from the majority of exoskeleton robots among both the research and commercial prototypes, REX features a completely self-supporting ability during training. This means it can maintain stability and balance without the reliance on crutches or external frame support. Its unique single-leg standing mode is particularly beneficial for stroke patients who lack independent standing ability, as it facilitates weight-bearing training on their affected leg. The specialized training program involved not only lower limb and balance training but also trunk posture correction and upper body motor control training with support from the REX (Figure 3, Table 4).

**Table 4:** The specialized training program on REX. PNF: proprioceptive neuromuscular facilitation.

Area of focus	Intervention
Sit-to-stand transition ability	Training for transferring from sitting to standing and standing to sitting with the help of the exoskeleton.
Muscle strength and proprioceptive input for the hemiplegic leg	Single-leg weight-bearing strengthening training on the affected side to enhance proprioceptive input with support from the exoskeleton; Training modes: lateral stepping, repetitive single-leg stepping, squatting training mode, and alternate stepping.
Trunk control ability	Dynamic and static trunk control training; Reach forward to the target with the hands clasped together (Bobath concept of handshake).
Walking ability	The therapist operates the exoskeleton to help patients with forward/backward walking training.
Upper limbs strength	Upper limbs (affected/unaffected side) resistance training in the diagonal direction (PNF concept).
Overall function for upper limbs	Throw and catch a ball; Boxing training; Basketball slaps.

Follow-up and outcome of interventions

After a three-week interval, the same set of evaluations was repeated, and the findings are outlined in Table 5. The Berg Balance Scale score improved to 54 after three weeks of treatment compared to pre-treatment, and the functional ambulation category scale (FAC) score also improved, indicating enhanced balance function and independent walking ability for the patient. Furthermore, there was an increase in the scores for both upper and lower limbs on the Fugl-Meyer rating scale; the lower limb score increased from 18 to 29, and the upper limb score increased from 43 to 59, indicating improvement in mobility and motor function of hemiplegic limbs. At the same time, the postural control scale for stroke patients (PASS) score showed a significant increase after three weeks of treatment compared to before, rising from 11 to 36. This indicates a marked improvement in the patient's ability to control their posture. 

Additionally, we conducted a follow-up with the patient six months after discharge. The patient is currently engaged in performing simple upper limb stretching exercises and unassisted walking training twice a day at home. He has achieved complete independence in activities of daily living. Moreover, he participates in daily leisurely walks with friends while walking his dog for entertainment. He expressed satisfaction with his balance and lower limb function.

**Table 5:** Outcome measures. FAC: functional ambulation category scale; PASS: the postural control scale for stroke patients; MMT-Grade 3: Full range of motion against gravity; MMT-Grade 4: Full range of motion against gravity with moderate assistance; the MCID of Berg Balance Score is 13.5; * means achieving the MCID of this assessment if the MCID is available. MCID: minimum clinically important difference.

	Pre-treatment	After treatment for three weeks
Berg Balance Score	3/56	54/56*
FAC score	0	3
Fugl-Meyer score	Upper limb 43	Upper limb 59*
	Lower limb 18	Lower limb 29*
PASS score	11	36*
Manual muscle testing (MMT) of knee extension	Grade 3	Grade 4

## Discussion

This case study found that after three weeks of wearable self-balancing exoskeleton robot training, the balance and lower limb motor ability on the affected side were significantly improved in an individual with stroke.

Stroke commonly causes somatosensory deficits and asymmetrical muscle strength decrease between bilateral limbs, resulting in trunk instability, imbalance, poor motor control, and even mobility-related falls [8]. The motor deficits also reduce the aerobic exercise ability through prolonged bed rest, and a significant decline in cardiopulmonary function adaptability after stroke leads to secondary cardiovascular problems, resulting in a vicious cycle of disability [5]. It is reported that simply getting out of bed can significantly increase heart rate, blood pressure, and oxygen saturation and improve conscious state [9]. Trunk exercise has been proven to have a considerable medium-term impact on subsequent tasks such as balance [10]. Therefore, early mobility [9] and postural control training [5] are all crucial (in aerobic and strength training levels, respectively) for stroke rehabilitation to improve the quality of life [11]. In this case report, the training modality adopted in wearable self-balancing exoskeleton robot has been preliminary proven particularly suitable for stroke patients with weak lower limb strength and trunk control, which allows severe patients to train in functional situations they would not otherwise be exposed to.

Proper postural control is imperative to maintain self-dynamic balance [12]. Self-dynamic balance refers to the inherent ability of the human body to transition between different postures without external force assistance. It is one of the prerequisites for patients to walk independently. In this study, the patient initially exhibited an inability to maintain balance in a standing position but eventually achieved level 2 balance in a standing position, indicating significant progress toward self-dynamic balance. The potential mechanism for patients' balance improvement may be that the robot provides feedback on weight support, repetitive movement, and proprioception during rehabilitation training [12]. Additionally, it helps maintain trunk stability and symmetry during mobility [13], which is conducive to enhancing motor control and coordination of patients' lower limbs [14].

Another attributing factor for the significant improvement is the robot’s ability to provide efficient and personalized doses. Brain plasticity plays a crucial role in motor recovery after stroke, involving neuronal reorganization, synaptic plasticity, axonal sprouting, limited neurogenesis, changes in neurotransmitter systems, and structural changes. From a rehabilitation perspective, repeated, high-dose training can help activate brain regions and reorganize networks to improve functional recovery [15,16]. Robots can provide guidance force and weight support [17] with the exoskeleton device, allowing personalized adjustment training intensity based on the severity of the participants. This creates a safe and simplified environment for generating repeatable normal gait pattern generation, giving it a distinct advantage compared with conventional rehabilitation therapy [18]. At present, a large number of studies have found that using rehabilitation robot training in stroke, subacute stage and chronic stages can obtain better therapeutic effects [19].

Furthermore, the therapist is able to free up their hands and reduce concerns about the risk of falls, allowing them to focus solely on the training. The patient experienced a sense of satisfaction and regained confidence as a result of overall functional performance improvement. He showed good adherence to the self-balancing exoskeleton robot and the training protocols, effectively illustrating the beneficial influence of contemporary technology on clinical rehabilitation practices.

## Conclusions

The self-balancing exoskeleton lower limb robot has shown great potential in the rehabilitation treatment of ischemic stroke patients, according to this case study. It can significantly enhance patients' postural stability and lower extremity mobility, thereby improving their overall quality of life. However, there is still further work that can be done in future research. For example, neurofunctional imaging tools can be employed to investigate the recovery mechanism behind exoskeleton-assisted rehabilitation. The optimal application population for self-balancing exoskeleton robots is also essential to discuss. It is important to acknowledge that the case report outcomes are preliminary, and further high-quality randomized controlled trials are imperative to confirm its effectiveness.

## References

[1] Handelzalts S, Kenner-Furman M, Gray G, Soroker N, Shani G, Melzer I (2019). Effects of perturbation-based balance training in subacute persons with stroke: a randomized controlled trial. Neurorehabil Neural Repair.

[2] Moore SA, Boyne P, Fulk G, Verheyden G, Fini NA (2022). Walk the talk: current evidence for walking recovery after stroke, future pathways and a mission for research and clinical practice. Stroke.

[3] Winstein CJ, Stein J, Arena R (2016). Guidelines for adult stroke rehabilitation and recovery: a guideline for healthcare professionals from the American Heart Association/American Stroke Association. Stroke.

[4] Teasell R, Salbach NM, Foley N (2020). Canadian stroke best practice recommendations: rehabilitation, recovery, and community participation following stroke. Part one: rehabilitation and recovery following stroke; 6th edition update 2019. Int J Stroke.

[5] Lo K, Stephenson M, Lockwood C (2017). Effectiveness of robotic assisted rehabilitation for mobility and functional ability in adult stroke patients: a systematic review. JBI Database System Rev Implement Rep.

[6] Kuo CY, Liu CW, Lai CH, Kang JH, Tseng SH, Su EC (2021). Prediction of robotic neurorehabilitation functional ambulatory outcome in patients with neurological disorders. J Neuroeng Rehabil.

[7] Poritz JM, Taylor HB, Francisco G, Chang SH (2020). User satisfaction with lower limb wearable robotic exoskeletons. Disabil Rehabil Assist Technol.

[8] Hu S, Wu G, Wu B, Du Z, Zhang Y (2022). Rehabilitative training paired with peripheral stimulation promotes motor recovery after ischemic cerebral stroke. Exp Neurol.

[9] Billinger SA, Arena R, Bernhardt J (2014). Physical activity and exercise recommendations for stroke survivors: a statement for healthcare professionals from the American Heart Association/American Stroke Association. Stroke.

[10] Souza DC, de Sales Santos M, da Silva Ribeiro NM, Maldonado IL (2019). Inpatient trunk exercises after recent stroke: an update meta-analysis of randomized controlled trials. NeuroRehabilitation.

[11] Hong E (2015). Comparison of quality of life according to community walking in stroke patients. J Phys Ther Sci.

[12] Liepert J, Bauder H, Wolfgang HR, Miltner WH, Taub E, Weiller C (2000). Treatment-induced cortical reorganization after stroke in humans. Stroke.

[13] Xie L, Yoon BH, Park C, You JS (2022). Optimal intervention timing for robotic-assisted gait training in hemiplegic stroke. Brain Sci.

[14] Mehrholz J, Thomas S, Elsner B (2017). Treadmill training and body weight support for walking after stroke. Cochrane Database Syst Rev.

[15] Yang C, Zhang T, Huang K, Xiong M, Liu H, Wang P, Zhang Y (2022). Increased both cortical activation and functional connectivity after transcranial direct current stimulation in patients with post-stroke: a functional near-infrared spectroscopy study. Front Psychiatry.

[16] Demers M, Varghese R, Winstein C (2022). Retrospective analysis of task-specific effects on brain activity after stroke: a pilot study. Front Hum Neurosci.

[17] Cherni Y, Hajizadeh M, Dal Maso F, Turpin NA (2021). Effects of body weight support and guidance force settings on muscle synergy during Lokomat walking. Eur J Appl Physiol.

[18] Chen SC, Kang JH, Peng CW, Hsu CC, Lin YN, Lai CH (2022). Adjustable parameters and the effectiveness of adjunct robot-assisted gait training in individuals with chronic stroke. Int J Environ Res Public Health.

[19] Meng G, Ma X, Chen P (2022). Effect of early integrated robot-assisted gait training on motor and balance in patients with acute ischemic stroke: a single-blinded randomized controlled trial. Ther Adv Neurol Disord.

